# Pathological Laughter: The First Manifestation of Intracerebral Haemorrhage Linked to Phenylephrine Overuse

**DOI:** 10.7759/cureus.63461

**Published:** 2024-06-29

**Authors:** David Prentice, Ferry Dharsono, Patricia Martinet

**Affiliations:** 1 Neurosciences, Perron Institute for Neurological and Translational Science, Nedlands, AUS; 2 Neuroradiology, Neurological Intervention and Imaging Service of Western Australia, Sir Charles Gairdner Hospital, Nedlands, AUS; 3 Internal Medicine, Fiona Stanely Hospital, Murdoch, AUS

**Keywords:** over-the-counter drugs, phenylepherine, reversible cerebral vasocontriction syndrome, pathological crying and laughter, intracranial hemorrage

## Abstract

A 42-year-old woman presented to the emergency department with sudden-onset uncontrollable laughter, ‘fou rire prodromique’ (prodrome of crazy laughter), and left leg weakness. Imaging revealed a right cerebral haemorrhage of the premotor cortex corresponding to the leg cortical representation. A history of excess phenylephrine use for sinusitis and migraine was subsequently obtained. The patient’s neurological recovery was good, enabling a return to her pre-stroke employment. The neurological causes of pathological laughter and the brain networks involved are discussed in this report. The role of sympathomimetics in the causation of cerebral haemorrhage potentially via the initiation of a reversible cerebral vasoconstriction syndrome is highlighted. The use of over-the-counter substances should be part of medication history in such cases.

## Introduction

The FDA advised of the association between phenylpropanolamine (PPA) and haemorrhagic strokes, and for the past two decades, no products containing PPA have been authorised in Australia [1]. Phenylephrine, a member of phenylethanolamines, has been linked to acute stroke occurrence in more than half a dozen cases and, however, remains available over the counter [2].

Phenylephrine is a potent vasoconstrictor and reversible cerebral vasoconstriction syndrome (RCVS) studies have found that women and migraine sufferers are at higher risk of intracranial haemorrhage [2,3]. In the case presented in this report, phenylephrine overuse in a middle-aged female with migraine history resulted in a cerebral haemorrhage that manifested as pathological laughter (PL), otherwise known as ‘fou rire prodromique’ [4]. Multiple neurological pathways are involved in laughter, such that PL can occur not only in pontine lesions and infarctions but also in cortical strokes.

Our patient was engaged in meditation at the time of onset of stroke signs, with her haemorrhage occurring in the premotor cortex. This case stresses the importance of including over-the-counter substances in a medication history, as they are often overlooked. The patient we present took 80 mg/day for at least six weeks prior to her presentation whereas the recommendation is a maximum dosage of 60 mg/day for a maximum of seven days. 

## Case presentation

A 42-year-old female was in a workplace meditation class when she developed a sudden onset of giggles and laughter that she recognised as inappropriate. It lasted only two minutes. At the time, she was seated upright at a desk and listening to soothing music as part of the meditation. Being extremely embarrassed, she tried to get up but noted a numbness in her left foot. On enquiry, this was not a tickling sensation. Left leg and arm weakness developed rapidly and she was hospitalised.

In the preceding weeks, she had used eight tablets per day of 10 mg per tablet strength of phenylephrine for sinusitis which was self-diagnosed. A history of classical migraine was diagnosed by her local doctor, but the sinus pain was different to those headaches. There was a paternal family history of stroke, diabetes and coronary artery disease. Past medical history included a bicuspid aortic valve found on investigation of an asymptomatic heart murmur. She also had mild scoliosis, conservatively managed. There was no history of alcohol or recreational drug use. She took no other prescribed or non-prescription medications; specifically had never used antidepressants, antihypertensives or hormonal therapy. No recent stress, trauma, or illness was elicited.

On ambulance arrival, examination of her vital signs were: temperature of 37 degrees, blood pressure of 149/106; heart rate was 86 beats per minute and regular. She remained afebrile and her blood pressure returned to normal (< 130/80) over the next 24 hours without treatment. Neurological examination findings were of left hemiparesis graded 0/5 power throughout, except for preserved finger flexion. Right-sided power was normal. There was bilateral lower limb hyperreflexia with Babinski responses. Sensation was intact. The remainder of the physical examination was normal, except for a soft systolic murmur.

Investigations

Investigations included a bedside ECG, midstream urine, and chest X-ray. Blood tests ordered in the emergency department and during her subsequent admission included full blood picture, urea and electrolytes, haemoglobin A1C, thyroid function, coagulation, beta-human chorionic gonadotropin (hCG), and autoimmune screen. All investigations were within normal limits.

**Figure 1:**
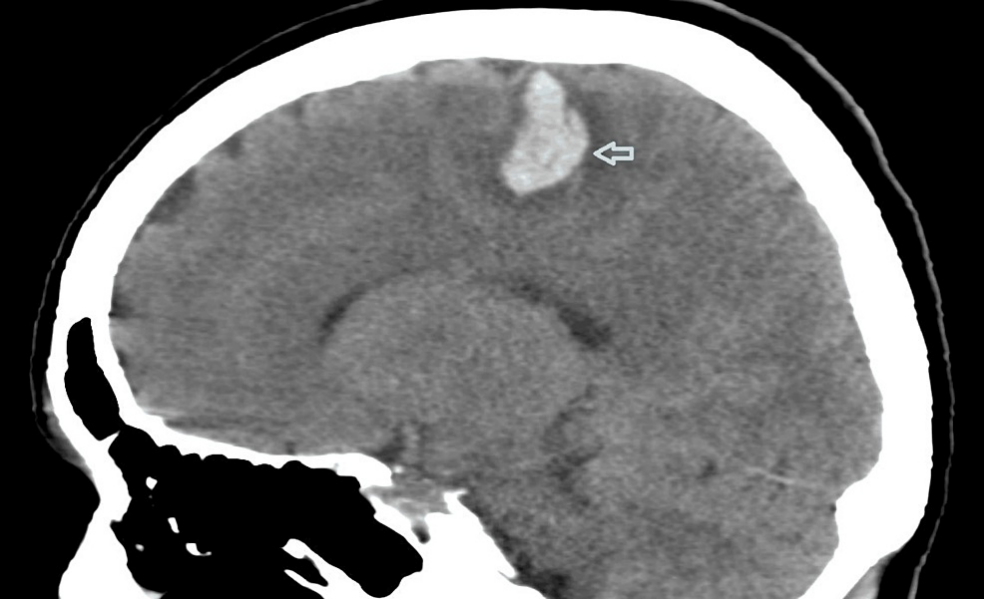
CT Head saggital view at admission shows parenchymal haematoma in right precentral gyrus and posterior aspect of right superior frontal gyrus

Neuroimaging included a computerised tomography (CT) head and angiogram. This revealed an acute cerebral haemorrhage involving the right superior frontal gyrus and abutting the pre-central gyrus, with surrounding oedema (Figure 1). A small amount of subarachnoid blood in sulci of the right frontal lobe was also reported. On the CT cerebral angiogram, there was no aneurysm, arterio-venous malformation, dissection, occlusion, or intravascular stenosis.

**Figure 2:**
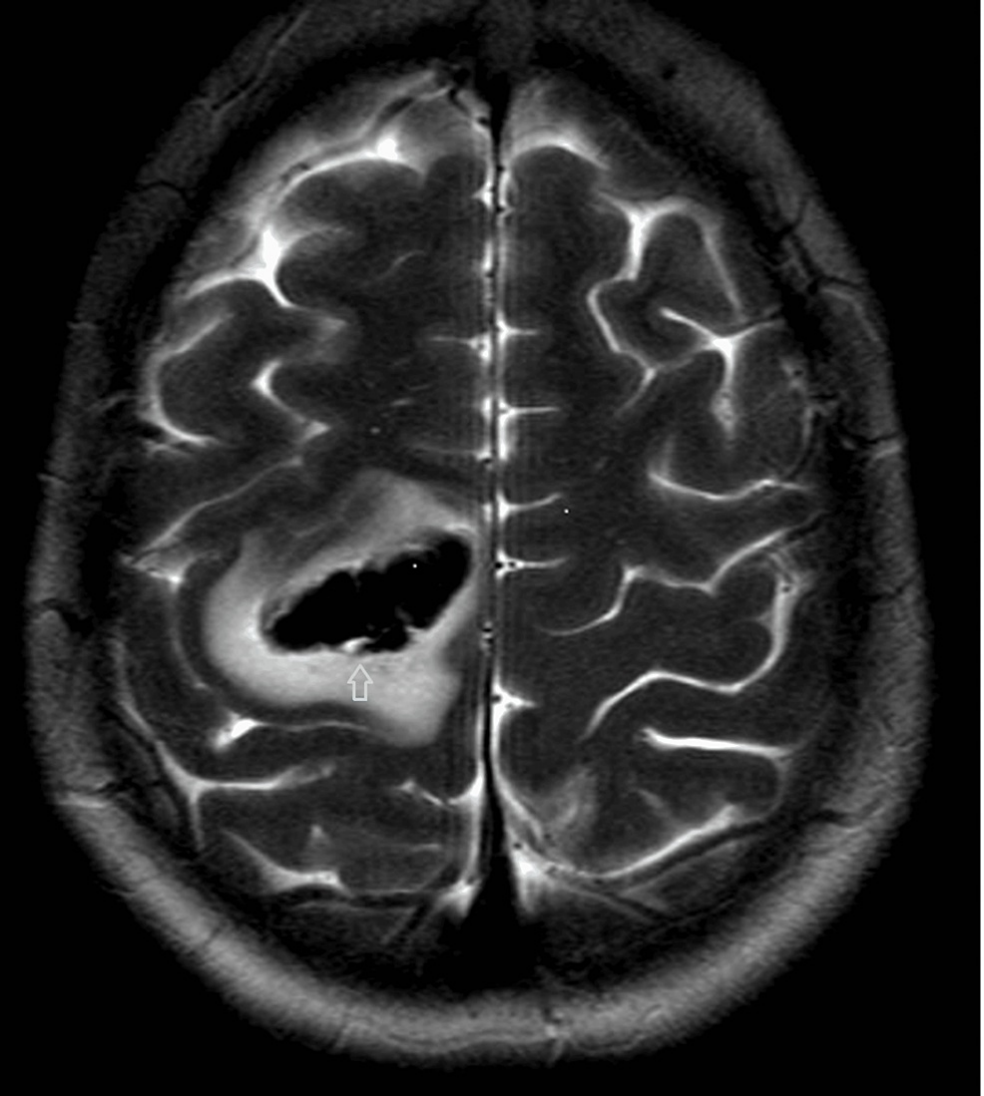
T2 axial MRI image on day 3 shows parenchymal haematoma in right precentral gyrus and posterior aspect of right superior frontal gyrus.

Magnetic resonance imaging (MRI) and MRI angiogram of her brain on day 3 showed an evolving haematoma in the right pre-central and superior frontal gyri with oedema (Figure 2) and no vascular malformation. The patient was offered a catheter cerebral angiogram but declined. No remote or punctate haemosiderin deposit was found elsewhere. Venous sinuses and cortical veins were unremarkable, with no thrombosis.

**Figure 3:**
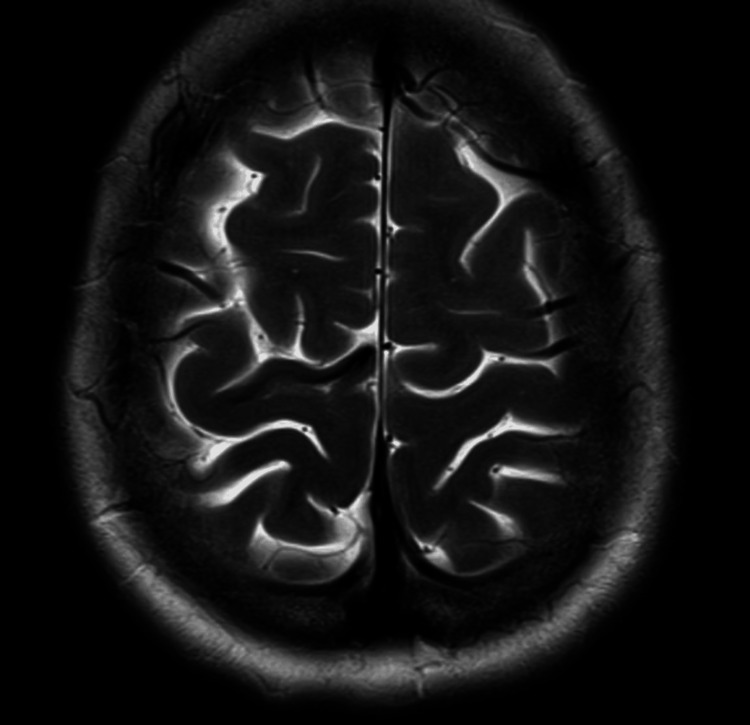
T2 axial MRI showing resolution of haemorrhage, two months post admission

A repeat MRI approximately two months later showed resolution of the haematoma and no evidence of any underlying mass lesion (Figure 3). A further repeat MRI 18 months post her intracerebral haemorrhage (ICH) showed resolution of the haematoma (identical to Figure 3) and no other causes for her haemorrhage. MRI and MRI cerebral angiogram eight years post presentation was normal.

Treatment

The patient was discharged from the rehabilitation unit 20 days post admission. Treatment consisted of in-patient rehabilitation therapies, with continued rehabilitation at home on discharge. Pharmacological treatment was solely paracetamol. Her blood pressure was monitored but systolic blood pressure remained consistently less than 130 mmHg and she did not require blood pressure lowering.

The patient made a complete recovery with minimal residual weakness in her left arm and foot. She reports inability to wear high heels as her only physical limitation. She returned to full-time employment within 18 months. Since cessation of phenylephrine, there has been no further intracerebral events.

## Discussion

We believe that the chronic, excessive use of phenylephrine led to the patient's intracerebral hemorrhage (ICH) with PL. We discuss the potential mechanisms for both.

Vasoconstrictive substances and ICH 

Overuse and misuse of sympathomimetic substances from cocaine to over-the-counter nasal decongestants have been associated with ICH [2,5]. It is postulated that prolonged vasoconstriction of the cerebral arteries leads to arterial necrosis with subsequent haemorrhage. Stoessl et al. demonstrated cerebral vasospasm associated with ICH in two first-time users of oral catecholaminergic medication [6]. Alternating narrowing and dilatation of the middle cerebral arteries was noted, which is identical to the findings seen in typical RCVS, commonly referred to as “sausage on a string” or “string of beads” [6].

Phenylephrine is an over-the-counter sympathomimetic drug for nasal decongestion. It works via its postsynaptic alpha-adrenergic receptor effect of constricting nasal blood vessels. Other oral decongestants such as PPA have been withdrawn due to association with haemorrhagic stroke [2]. Both nasal and oral preparations, including those containing pseudoephedrine, have been implicated in stroke and cardiovascular effects. The association between over-the-counter sympathomimetics and RCVS is well documented [6]. Furthermore, almost half the patients in Cantu et al.'s study with over-the-counter sympathomimetic use and stroke had beading in the carotid or vertebrobasilar arteries or branches [3].

Tark et al. reported the case of a 59-year-old female with a thunderclap headache followed by hemiparesis and aphasia [2]. She had a similar history to the current patient and had been taking phenylephrine for sinusitis during the preceding month. A left frontal ICH was diagnosed on a brain CT scan. The authors reviewed the literature, discovering seven cases of stroke with phenylephrine use, leading to their postulation of a causal relationship between phenylephrine and ICH. Our case is supportive of this conclusion, especially given the absence of any underlying abnormalities detected in blood work and imaging, together with our patient’s unremarkable past medical history.

Although no cerebral vasoconstriction was demonstrated in our patient’s CT head angiography to justify a diagnosis of RCVS, it seems likely that her ICH was a consequence of RCVS, in the context of overuse of phenylephrine. Vasoactive drugs such as phenylephrine are the most common trigger cited for RCVS [7].

PL and stroke

Normal laughter can be invoked by humour, tickling and social cues (bonding, agreement, affection) [8]. PL has no strict definition but it is laughter which is mirthless, irrepressible and inappropriate to the person’s mood and state of mind. It has been described in a number of conditions including cerebrovascular disease, motor neurone disease, Parkinson’s, multiple sclerosis, cerebral tumours, epilepsy, and traumatic brain injury [8]. 'Fou rire prodromique' was first coined in 1903 to describe the rare condition that is acute onset PL in the context of a stroke. In the literature, the term has been used in the context of PL presaging a stroke and as a symptom of an acute stroke [9,10].

The neuropathology of PL involves multiple areas of the brain. A 2021 publication used lesion network symptom mapping to identify two important networks being involved in PL, namely (i) positive connectivity to the cingulate and temporomesial cortices, striatum, hypothalamus, mesencephalon and pons, and (ii) negative connectivity to the primary motor and sensory cortices [11]. The loss of the inhibitory effect of these cortical neuronal networks releases the emotional facial expression pathways, resulting in PL. Bilateral corticospinal tracts inhibit the facial-respiratory pontine laughter centre [12].

While rare, PL is more widely presented in the literature as an association with pontine infarction and other pontine pathologies [13]. Lesions involved in PL are found in the pons’ base bilaterally, left thalamus and para-hippocampal gyrus, left lenticular and caudate nucleus, and area perfused by the right middle cerebral artery [8]. Dulamea et al. described a 59-year-old man who had recurrent left carotid transient ischemic attacks characterised by aphasia, hemiparesis, and PL [13]. Multiple electroencephalograms (EEGs) were normal, ruling out gelastic seizures. In our patient, gelastic epilepsy was considered unlikely due to the absence of a hypothalamic hamartoma, the brief duration of PL, and the absence of neuropathological past medical history. However, we cannot rule out that our patient experienced a gelastic seizure at the onset of her bleed.

A literature search revealed only three other cases of 'fou rire prodromique’ in association with middle cerebral artery strokes and all were left-sided [13,14]. To the best of our knowledge, there are no other case reports of a cortical ICH with PL. Interestingly, our patient is the only one with a right-sided lesion as per our search. An explanation for our patient’s presentation may be related to RCVS. It has been shown by arterial spin-labelling perfusion studies that cerebral artery watershed areas in RCVS can develop ischemia [15]. It may be argued that vasoconstriction induced by phenylephrine interrupted the negative connectivity pathway. The resulting haemorrhage and hypoxic dysfunction in that area presented as PL. Seizures are known to occur after a cortical haemorrhage. An alternative explanation might be an excitation of the supplementary motor area immediately post haemorrhage. PL has been induced by electrical stimulation of this cortical region [16]. However, the rarity of PL with strokes is difficult to explain. Perhaps the individual’s activity engagement and mind state at the time is relevant.

## Conclusions

PL is rare in strokes and is more common in pontine infarcts. The neurophysiology of laughter is complex and involves at least two extensive brain networks, including the involvement of the cortices. Misuse of nasal decongestants can be associated with cerebral haemorrhage and infarction. A thorough medication history should expressly elicit the dosage of over-the-counter substances being consumed. Reversible vasoconstriction underlies the cause of ICH in vasoactive drugs such as phenylephrine.
